# Resistance-minimising strategies for introducing a novel antibiotic for gonorrhoea treatment: a mathematical modelling study

**DOI:** 10.1016/S2666-5247(23)00145-3

**Published:** 2023-08-21

**Authors:** Emily Reichert, Reza Yaesoubi, Minttu M Rönn, Thomas L Gift, Joshua A Salomon, Yonatan H Grad

**Affiliations:** Department of Immunology and Infectious Diseases, Harvard TH Chan School of Public Health, Boston, MA, USA; Department of Health Policy and Management, Yale School of Public Health, New Haven, CT, USA; Department of Global Health and Population, Harvard TH Chan School of Public Health, Boston, MA, USA; Division of STD Prevention, Centers for Disease Control and Prevention, Atlanta, GA, USA; Department of Health Policy, Stanford University School of Medicine, Stanford, CA, USA; Department of Immunology and Infectious Diseases, Harvard TH Chan School of Public Health, Boston, MA, USA

## Abstract

**Background:**

Gonorrhoea is a highly prevalent sexually transmitted infection and an urgent public health concern because of increasing antibiotic resistance in *Neisseria gonorrhoeae*. Only ceftriaxone remains as the recommended treatment in the USA. With the prospect of new anti-gonococcal antibiotics being approved, we aimed to evaluate how to deploy a new drug to maximise its clinically useful lifespan.

**Methods:**

We used a compartmental model of gonorrhoea transmission in a US population of men who have sex with men (MSM) to compare strategies for introducing a new antibiotic for gonorrhoea treatment. The MSM population was stratified into three sexual activity groups (low, intermediate, and high) characterised by annual rates of partner change. The four introduction strategies tested were: (1) random 50–50 allocation, where each treatment-seeking infected individual had a 50% probability of receiving either drug A (current drug; a ceftriaxone-like antibiotic) or drug B (a new antibiotic), effective at time 0; (2) combination therapy of both the current drug and the new antibiotic; (3) reserve strategy, by which the new antibiotic was held in reserve until the current therapy reached a 5% threshold prevalence of resistance; and (4) gradual switch, or the gradual introduction of the new drug until random 50–50 allocation was reached. The primary outcome of interest was the time until 5% prevalence of resistance to each of the drugs (the new drug and the current ceftriaxone-like antibiotic); sensitivity of the primary outcome to the properties of the new antibiotic, specifically the probability of resistance emergence after treatment and the fitness costs of resistance, was explored. Secondary outcomes included the time to a 1% resistance threshold for each drug, as well as population-level prevalence, mean and range annual incidence, and the cumulative number of incident gonococcal infections.

**Findings:**

Under baseline model conditions, a 5% prevalence of resistance to each of drugs A and B was reached within 13·9 years with the reserve strategy, 18·2 years with the gradual switch strategy, 19·2 years with the random 50–50 allocation strategy, and 19·9 years with the combination therapy strategy. The reserve strategy was consistently inferior for mitigating antibiotic resistance under the parameter space explored and was increasingly outperformed by the other strategies as the probability of de novo resistance emergence decreased and as the fitness costs associated with resistance increased. Combination therapy tended to prolong the development of antibiotic resistance and minimise the number of annual gonococcal infections (under baseline model conditions, mean number of incident infections per year 178 641 [range 177 998–181 731] with combination therapy, 180 084 [178 011–184 405] with the reserve strategy).

**Interpretation:**

Our study argues for rapid introduction of new anti-gonococcal antibiotics, recognising that the feasibility of each strategy must incorporate cost, safety, and other practical concerns. The analyses should be revisited once robust estimates of key parameters—ie, the likelihood of emergence of resistance and fitness costs of resistance for the new antibiotic—are available.

**Funding:**

US Centers for Disease Control and Prevention, National Institute of Allergy and Infectious Diseases.

## Introduction

Increasing antibiotic resistance in *Neisseria gonorrhoeae* poses an urgent clinical and public health threat. Only one antibiotic—ceftriaxone, an extended spectrum cephalosporin—remains recommended by the US Centers for Disease Control and Prevention guidelines for empirical treatment of gonorrhoea in the USA,^1^ underscoring the need to develop new antibiotics to treat this highly prevalent sexually transmitted infection. Two promising, first-in-class candidates currently in phase 3 clinical trials are zoliflodacin and gepotidacin. In phase 2 trials, both demonstrated cure rates of 96% for urogenital infection; cure rates for pharyngeal infection, which has been historically harder to cure, were lower.^2,3^ Given the scarcity of tools remaining for gonorrhoea treatment, it is imperative to deploy new antibiotics in a way that prolongs their clinical effectiveness.

The question of how best to use multiple effective antibiotics with the aim of minimising population-level resistance has been explored in mathematical modelling studies considering a range of strategies: (1) random allocation of multiple drugs, (2) combination therapy, (3) holding in reserve a second-line drug until prevalence of resistance to the first-line drug reaches a defined threshold, (4) cycling treatment, and (5) targeted (directed) treatment, where a second-line drug is given only to patients who are non-responsive to the first.^4-11^ One pervasive finding across these studies is that antibiotic cycling, or the rotation of the antibiotic used as first-line therapy according to a set schedule, is inferior for mitigating antibiotic resistance; however, among alternative approaches the optimal strategy varies by study setting and pathogen of interest. Although one study focused specifically on gonorrhoea,^11^ none explored how and when to optimally introduce a new antibiotic.

Historically, US guidelines for gonorrhoea treatment have followed several strategies. Multiple options enabled providers to choose antibiotics during the 1990s. Increasing resistance then led to combination therapy of ceftriaxone plus azithromycin; however, rising azithromycin resistance in 2020 prompted a return to single-drug treatment with ceftriaxone.^12^ The question of how to best allocate multiple antibiotics will once again become relevant if zoliflodacin, gepotidacin, or another candidate gains US Food and Drug Administration (FDA) approval. Therefore, this study aimed to compare the effect of introduction strategies for a novel antibiotic on resistance of *N gonorrhoeae* in a US population of men who have sex with men (MSM) using a mathematical model of gonorrhoea transmission. We focused on MSM as approximately a third of US cases occur within this population^13^ and, because of the historic appearance of resistance to antibiotics such as ciprofloxacin within this population,^14^ gonorrhoea transmission models characterising US MSM have been well researched.^15-17^

## Methods

### Model overview

**Figure 1:**
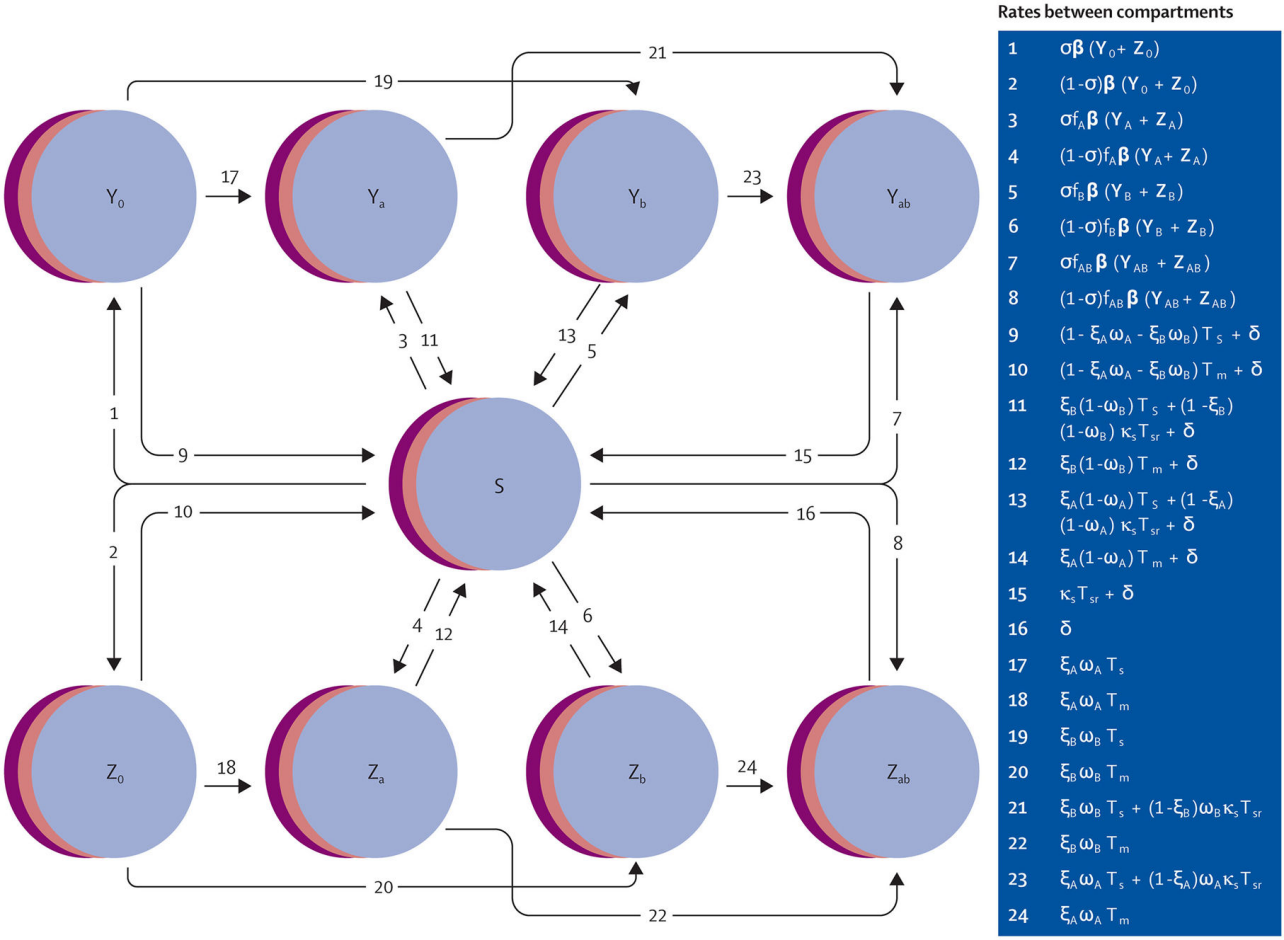
Schematic of gonorrhoea transmission model Infections are further stratified by resistance profile, where 0=susceptible, a=resistant to drug A, b=resistant to drug B, and ab=resistant to both drugs. Overlapping discs for all compartments represent the model’s stratification into three sexual activity groups: low, intermediate, and high. Arrows depict rates between compartments and would be multiplied by the compartment from which they flow to generate the model’s set of differential equations (appendix, pp 8-9). Individuals can also enter and exit the population at rate ρ (arrows not shown). Rates shown here apply to random 50–50 allocation, reserve, and gradual switch strategies; rates for combination treatment vary slightly and are shown in the appendix (p 9). A transition from a drug-susceptible infection (Y_0_, Z_0_) to a dual resistant infection (Y_AB_, Z_AB_) is only possible under combination therapy (arrows not shown). Definitions of all parameters used in rate equations are in table 1. S=susceptible. Y=symptomatic infection. Z=asymptomatic Infection.

We modified the susceptible–infectious–susceptible deterministic compartmental gonorrhoea transmission model described in a previous study (figure 1, appendix pp 7-10).^17^ Briefly, our model consisted of an MSM population stratified into three sexual activity groups (low, intermediate, and high) characterised by annual rates of partner change. Sexual activity group was fixed for each individual, but individuals from different groups interacted with mixing parameter ε. Individuals aged into and out of the sexually active population at rate ρ, contributing for 20 years on average. Infected individuals could recover spontaneously or through antibiotic treatment. For those who sought treatment, one could receive drug A (ceftriaxone-like antibiotic) or drug B (new antibiotic), or both. Infections were stratified by symptomatic (Y) versus asymptomatic (Z) status and by resistance profile, where each infection could be resistant to drug A, drug B, neither, or both. We use the term individual only to help conceptualise the movement of this population between model compartments. No ethical approval was required for this modelling study.

### Model parameterisation

Model parameters were determined from the scientific literature or model calibration using maximum likelihood estimations (table 1). We calibrated our model to a beta distribution [Beta(α=59·4, β=1919·2)] with mean 3·0% gonorrhoea prevalence (variance 1·47 × 10^−5^) at equilibrium, based on recent estimates in MSM.^13,21,22^ Model simulations for calibration were run for a drug A-only model over 2 years and parameterised using the R package bbmle.^23^

Antimicrobial susceptibility surveillance data from the Gonococcal Isolate Surveillance Project informed a conservative estimate of prevalence of resistance to the ceftriaxone-like drug (0·01%) at time 0.^12^ Relevant properties of ceftriaxone, including the probability of de novo resistance (ω_A_) and the fitness cost associated with resistance (1–f_A_), were inferred from previous studies.^17^ We used the term drug A to emphasise that we were modelling a ceftriaxone-like drug, as its parameters are only informed estimates. All gonococcal infections were presumed susceptible to the novel antibiotic, drug B, at time 0. Baseline properties of drug B, including the probability of emergence of de novo resistance upon treatment (ω_B_) and the associated fitness cost (1–f_B_), were partly informed by the few data points from phase 2 clinical trials for gepotidacin and zoliflodacin. The baseline probability of de novo resistance for drug B was greater than for drug A given gepotidacin’s trial results suggesting resistance upon treatment, observed in two of 69 patients, is more common than with ceftriaxone.^3^ The baseline relative fitness of strains resistant to drug B was lower than for drug A, in keeping with reports that *gyrB* mutations associated with zoliflodacin resistance incur a substantial fitness cost.^24^ However, absolute parameter estimates were assumed and widely explored in the sensitivity analysis, partly to present results useful for any newly approved antibiotic. We assumed resistance mechanisms to drugs A and B were independent, as both zoliflodacin and gepotidacin are first-in-class candidates (of spiropyrimidinetriones and triazaacenaphthylenes, respectively) that target gyrase and topoisomerase, whereas the third-generation cephalosporin ceftriaxone targets penicillin-binding proteins.^2,3^

### Antibiotic introduction strategies

We compared four different strategies for the deployment of drug B into the population. (1) Random 50–50 allocation: each treatment-seeking infected individual has a 50% probability of receiving either drug A or drug B, effective at time 0. (2) Combination therapy: each treatment-seeking infected individual receives both drug A and drug B, effective at time 0. (3) Reserve strategy: each treatment-seeking infected individual receives drug A until a 5% prevalence of resistance is reached in the population, at which point drug B is introduced with a sigmoid growth function until it is used for 100% of presenting cases. (4) Gradual switch: each treatment-seeking infected individual receives drug A at time 0, but drug B is gradually introduced into the population until complete random 50–50 allocation is reached within 9·5 years (midpoint 4 years).

A threshold of 5% resistance prevalence was used to trigger the switch from drug A to B under the reserve strategy, as this constitutes WHO’s threshold for changing treatment recommendations.^25^ We incorporated a gradual switch strategy into our analysis to depict a more realistic introduction scenario for a first-in-class antibiotic, assuming factors such as clinician hesitancy, drug supply, and distribution issues might delay uptake. For both the reserve and gradual switch strategies, the probabilities of receiving drugs A and B (ξ_A_, ξ_B_) for treatment-seeking individuals were not constant but updated throughout the model using sigmoid functions (appendix p 2). Antibiotic cycling was not considered in this analysis because of the aforementioned evidence against its benefit.

Of note, the above scenarios describe only the allocation of an individual’s initial course of treatment. Symptomatic individuals prescribed a drug that was ineffective against their infection could seek retreatment at rate T_sr_ with probability k_s_, at which point they received the alternative antibiotic, incorporating a targeted element into all approaches. An exception was assumed for symptomatic individuals with dual-resistant infection; these cases were retreated with a last-resort antibiotic external to our model for which we assumed complete efficacy and did not monitor resistance trends.

### Model implementation

The base model was initialised with the equilibrium prevalence of gonorrhoea achieved via calibration (3·0%) and simulated over 40 years using the R package deSolve version 1.34.^26^ For the primary outcome of interest, we compared the time until each of drugs A and B lost clinical effectiveness for empiric use, defined by reaching a 5% prevalence of resistance among gonococcal infections. We defined this time to loss outcome (T_L_) by the maximum of (1) the time to 5% resistance to drug A and (2) the time to 5% resistance to drug B. Secondary outcomes explored include the time to a 1% resistance threshold for each drug, as well as population-level prevalence, mean and range annual incidence, and the cumulative number of incident gonococcal infections.

Because baseline parameters describing the properties of the novel drug B were assumed, we performed sensitivity analyses to compare strategies across a large parameter space for both the probability of resistance emergence upon treatment with drug B (ω_B_), and the relative fitness of infections resistant to drug B (f_B_). Since parameters are not known with certainty for drug A (ceftriaxone-like antibiotic), we reran the sensitivity analysis over 100 years exploring drug B’s properties under two alternate scenarios for drug A: (1) fitness cost for resistant strains (1–f_A_) increased to 0·10, and (2) the probability of de novo resistance (ω_A_) increased to 10^−4^. Finally, to broadly assess the effect of model parameterisation, we recalibrated the model and assessed the primary time-to-loss outcome under two alternative targets: 1·5% and 6·0% mean gonorrhoea prevalence at baseline. All code needed to run the model and produce numeric output and figures is available online.

### Role of the funding source

The US Centers for Disease Control and Prevention contributed to study design and writing of the report. The National Institute of Allergy and Infectious Diseases had no role in study design, data collection, data analysis, data interpretation, or writing of the report.

## Results

The transmission model projected that continued monotherapy with drug A (a ceftriaxone-like antibiotic) for gonorrhoea treatment would result in a 5% prevalence of resistance warranting new empirical treatment guidelines within 6·5 years in our population (table 2; appendix p 3). Assuming introduction of the novel antibiotic B, with baseline parameters for its rate of de-novo resistance emergence and associated fitness cost, the time to loss (ie, a 5% resistance threshold) of drug A ranged from 6·5 to 19·9 years depending on the introduction strategy, whereas drug B’s clinical effectiveness ranged between 13·9 and 19·9 years (table 2; appendix p 3).

Combination therapy maximised the time until 5% resistance was met for each of the drugs (T_L_=19·9 years); random 50–50 allocation was a close alternative, reducing the time until the drugs were lost to T_L_=19·2 years (table 2; appendix p 3). By contrast, the reserve strategy led to a faster build-up of infections resistant to drug A; a 5% prevalence of resistance to drug A was present within 6·5 years. This facilitated an earlier emergence of dual resistance and resulted in the shortest combined empirical lifespan of the drugs for the reserve strategy (T=13·9 years) relative to alternative approaches.

Combination therapy also minimised the mean annual number of incident gonococcal infections up until T_L_ (table 2). Except for the reserve strategy, every strategy involving the introduction of drug B saw dual resistant strains rise to comprise more than 99% of infections after the treatments were lost given sufficient time and no alternative antibiotics; this led to the model reequilibrating at a gonorrhoea prevalence approximately three times that at baseline (appendix p 3). Under the reserve strategy, because the overlap of drug A and drug B’s use was minimised, strains resistant to drug B only took over following T_L_. However, if combination therapy was enacted after the loss of drug B at 13·9 years, dual resistant strains quickly rose to make up more than 99% of infections (appendix p 3). Although the reserve strategy minimised time to loss T_L_, it in turn maximised the incidence of gonococcal infection over time (appendix p 4).

**Figure 2:**
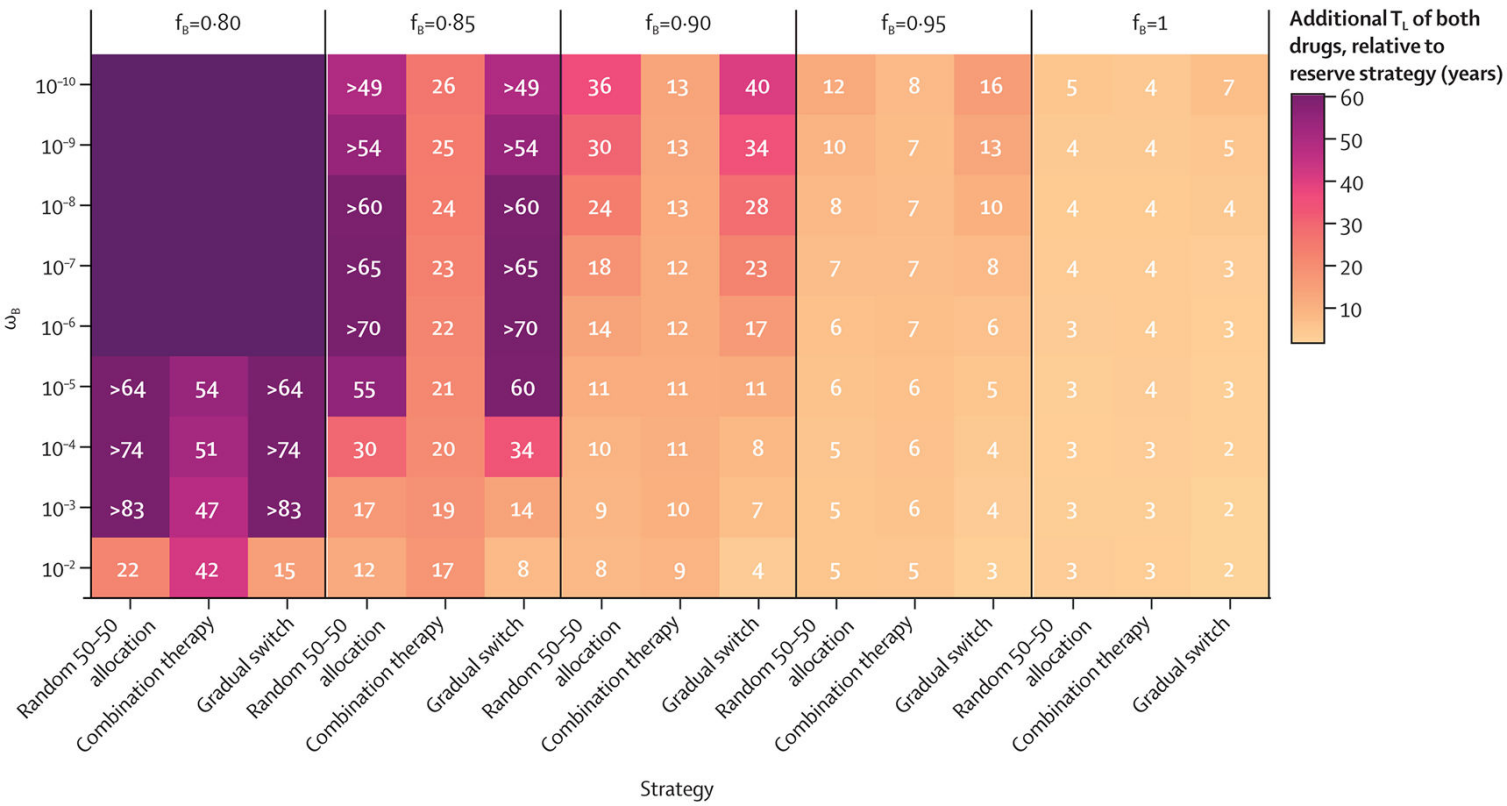
Additional T_L_ (in years) of each of drugs A (ceftriaxone-like) and B (new antibiotic) by strategy relative to that of the reserve strategy (T_L_ strategy–T_L_ reserve), based on properties of a new antibiotic B Strategies (x-axis) were compared over a range of plausible parameter values for ω_B_ (y-axis) and f_B_ (vertical facets). These properties were held constant for drug A: ω_A_=0·98 and f_A_=10^−8^. The model run time was extended to 100 years because some parameter sets increased the lifespan of available antibiotics to more than 40 years for all strategies. If the lifespan of the drugs extended beyond 100 years, that strategy’s results are shown either in relative terms for comparison or with an unlabelled dark purple tile, if no strategies on the x-axis had a defined T_L_ under that parameter set for drug B. f_B_=fitness of drug B resistant strains relative to susceptible bacteria. T_L_=time in years until both drugs A and B hit their 5% resistance thresholds, warranting new treatment recommendations. ω_B_=probability of emergence of resistance upon treatment with drug B.

Model simulations were then run for 100 years over a larger, two-dimensional parameter space for the properties of new drug B. The probability of emergence of resistance after treatment with drug B (ω_B_) varied from 10^−2^ to 10^−10^, and the fitness cost associated with resistance (1–f_B_) varied from 0 to 0·20. Assuming baseline parameters for drug A, keeping the novel antibiotic B in reserve until drug A’s failure was not favourable under any scenario as this strategy minimised the time until each of the drugs were lost for empiric use (T_L_; figure 2). The combined lifespan of the drugs under the reserve strategy lagged behind alternative approaches by 2–3 years if resistance to drug B was likely to emerge (ω_B_≥10^−4^) and spread (f_B_=1). Altering drug B’s characteristics to make the emergence and spread of resistance more unlikely resulted in the reserve strategy performing worse by greater margins compared with the alternatives (figure 2).

**Figure 3:**
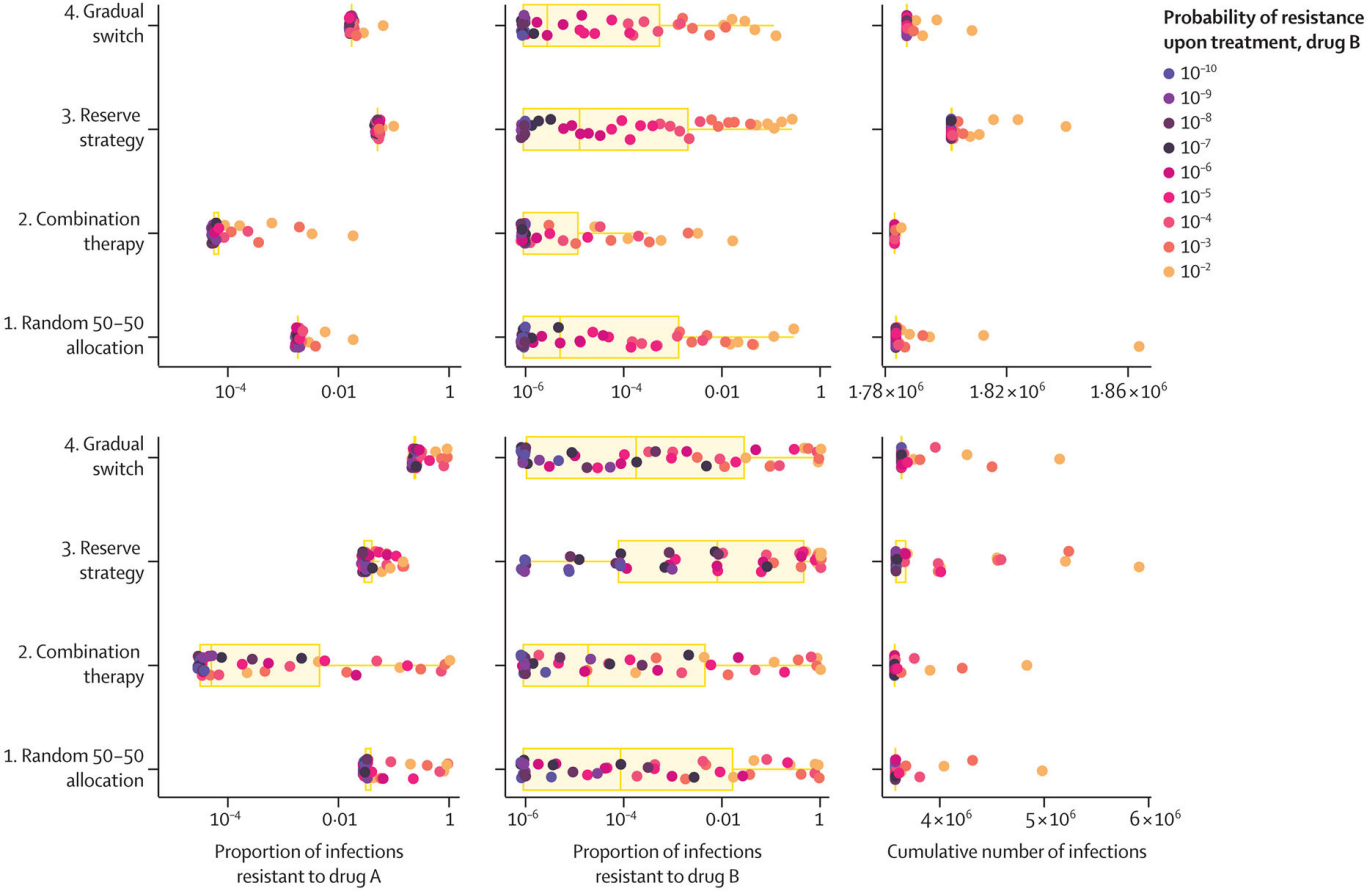
Distribution of antibiotic resistance to drugs A and B and the cumulative number of infections at years 10 and 20, by introduction strategy Each point represents the outcome from a different model run over one of 45 parameter sets for the relative fitness of bacteria resistant to drug B (f_B_ =0·80–1) and the probability of resistance upon treatment (ω_B_=10^−10^−10^−2^). Points are coloured by drug B’s probability of resistance upon treatment. Boxplots summarise the distribution of these outcomes across the 45 model runs by introduction strategy. These properties were held constant at f_A_=0·98 and ω_A_ =10^−8^ for drug A.

We then explored the within-strategy and between-strategy distributions of the prevalence of infections resistant to drug A and drug B, and the cumulative number of infections at two points in time (figure 3) for the same 45 potential combinations of parameters for drug B. The median proportion of infections resistant to drug A ranged from 0·006% (range 0·006–1·82) with combination therapy to 5·08% (5·08–10·0) with the reserve strategy at 10 years, and from 0·005% (range 0·003–98·7) with combination therapy to 23·4% (23·4–98·2) with the gradual switch strategy at 20 years. For drug B, the median proportion of resistant infections was 0·001% or less at 10 years for all strategies but ranged from 0·002% (range 0·000–98·7) with combination therapy to 0·80% (0·000–99·9) with the reserve strategy at 20 years.

To account for uncertainty in the parameter estimates for ceftriaxone, we evaluated two scenarios: (1) fitness cost for resistant strains (1–f_A_) increased to 0·10, and (2) probability of de novo resistance (ω_A_) increased to 10^−4^. Although absolute estimates varied, the additional time to loss of each of drugs A and B (T_L_) relative to that of the reserve strategy remained positive across all introduction strategies and drug B parameterisations (appendix p 5). The time by which other strategies extended the empirical use of drugs A and B relative to the reserve strategy generally increased in magnitude as both the probability of resistance emergence and the relative fitness of resistant strains decreased.

Finally, we recalibrated the model to achieve a target of 1·5% and 6·0% mean gonorrhoea prevalence at baseline (half and two times our initial assumption, respectively) and evaluated the primary time-to-loss outcome for each introduction strategy under this new model parameterisation (appendix p 6). Relative results were consistent with those previously observed.

## Discussion

This study showed that in a model of gonorrhoea transmission in a population representative of US MSM, among strategies to introduce a new antibiotic with the aim of slowing the spread of resistance, reserving the novel antibiotic until substantial resistance to the current first-line drug has arisen is inferior to strategies that introduce the novel antibiotic earlier. The effect of introducing the novel drug once it becomes available varies depending on its parameters; as resistance to drug B becomes less likely to emerge and more costly, the reserve strategy is increasingly outperformed by alternative strategies in terms of extending the clinical effectiveness of the available antibiotics.

In concordance with previous studies, no single introduction strategy for a new antibiotic targeting *N gonorrhoeae* proves robustly optimal in the long term.^4,6,9,10^ Under baseline assumptions, combination therapy maximised the clinical utility of the antibiotics and minimised average annual infections, with random 50–50 allocation a close second. Across all parameters explored for drug B, combination therapy also minimised cumulative infections at 10 and 20 years on average. Although the eventual emergence of dual resistant gonorrhoea strains made combination therapy comparable to other strategies long term, its benefits were seen most starkly in the short term, as it delayed emergence of resistant gonococcal strains. As the probability of resistance after drug B treatment decreased, other strategies became more favourable than combination therapy in maximising the drugs’ combined lifespan. This observation indicates that if resistance acquisition is already unlikely, combination therapy loses some advantage. For example, if FDA approval precludes antibiotic candidates with more than 10^−7^ probability of resistance emergence, the random 50–50 allocation and gradual switch approaches are consistently similar or superior to combination therapy.

Substantial between-strategy variation was found in the proportion of infections resistant to drug A at 10 and 20 years, despite drug A’s fixed parameters. This finding suggests that the introduction strategy used for drug B has consequences on resistance trends to drug A.

Our analyses focused on the time to loss of the drugs for empirical treatment (T_L_). Interpretation of results after T_L_ warrants caution. For example, the reserve strategy might initially appear attractive (appendix p 3), as it precludes the takeover of dual resistant strains. After the loss of drug B at 13·9 years, combination therapy could be enacted to successfully treat cases resistant to drug B only, but only for a short time until dual resistance takes over. Short-term model outcomes are also more relevant than long-term projections, as new tools for gonorrhoea management and prevention (eg, rapid antimicrobial susceptibility diagnostics and vaccines) in development might affect *N gonorrhoeae*’s dynamics. Of note, the reserve strategy lengthened the projected time between the loss of drugs A and B; although not inherently clinically relevant, having this lag might be more palatable to medical providers than losing both drugs in rapid succession.

Other factors beyond those explored here inform how to best deploy a new antibiotic. The cost of a novel therapeutic is a major factor in its use, as are safety, tolerability, allergy, mode and duration of administration, and drug-interaction concerns. Our analysis is best interpreted as an exploration of the ideal antibiotic introduction strategy for deterring resistance in *N gonorrhoeae* assuming that other practical concerns are not prohibitive (and keeping in mind key model limitations). For example, through exploring combination therapy, we assumed that this option is clinically safe and effective—ie, that drugs A and B have compatible pharmacokinetics, no antagonistic effects, and using a therapeutic dose of both in combination is non-toxic. The feasibility and effectiveness of combination therapy relies on these assumptions. A disparity in the pharmacokinetics of ceftriaxone and azithromycin is one hypothesis for why this combination therapy failed—azithromycin’s long elimination half-life compared with ceftriaxone could leave patients exposed to only a sub-inhibitory azithromycin concentration, an environment selective for resistance given recurrent gonococcal infection.^27^ Once cost and safety concerns are incorporated, random 50–50 allocation might be the most competitive choice as it proved only slightly less effective than combination therapy in reducing the burden of infection and resistance, on average, but is likely to minimise cost and risk of adverse events relative to combination therapy.

We were further limited in our study by ignoring bystander selection, or selection for resistance caused by gonorrhoea-infected individuals receiving antibiotics for other indications. The relative importance of bystander compared to direct selection for resistance in *N gonorrhoeae* is still unclear, and further research is needed to clarify its extent.^27^ Ceftriaxone is used to treat a variety of bacterial infections; evidence that bystander selection is a major driver of *N gonorrhoeae* resistance would support restricting use of a novel antibiotic to gonorrhoea indications. We also did not consider the possible importation of drug resistant strains to this US population from other countries with differing treatment policies.

Many of the model parameters describing gonorrhoea’s natural history and transmission are impossible to measure empirically, so we estimated these values with maximum likelihood estimation, an optimisation procedure that here used a single equilibrium prevalence target for model calibration. Prevalence estimates—of gonorrhoea as well as ceftriaxone-resistant strains—are potentially underestimated because of undetected asymptomatic infections. Further, estimating multiple parameters using a single prevalence estimate for calibration suggests that individual parameters would vary under alternative calibration targets or procedures.

Baseline assumptions for the characteristics of drug B were based on few phase 2 trial data for zoliflodacin and gepotidacin. Because of parameter uncertainty and our aim to present results applicable for any novel drug, we varied these parameters widely in sensitivity analysis. We also assumed no resistance to the novel antibiotic existed before its introduction, which might not be accurate. Mutations in *gyrB* associated with zoliflodacin resistance were found at a 0% prevalence in a large *N gonorrhoeae* genome database, but mutations associated with gepotidacin resistance appeared in isolates resistant to tetracycline, ciprofloxacin, and penicillin, suggesting that gepotidacin resistance might be more easily acquired in a subset of infections.^28,29^ Absolute estimates of the time to 5% resistance prevalence for both drugs should therefore be interpreted with caution and not extrapolated; results are best suited for relative interpretation within our population of interest, consistent with our aim of comparing the various introduction strategies.

It is key to acknowledge that our model focuses specifically on the MSM population; however, trends in gonorrhoea resistance in MSM in the USA have historically been forewarnings for resistance trends in the general population.^13,14^ The model does not differentiate infections by anatomical site, which might be relevant given the drug B candidates’ variable efficacy in curing non-urogenital infection.^2,3^ We also assumed uniform screening and treatment-seeking behaviour across MSM, by symptom status.

Overall, the steady accumulation of antibiotic resistance in *N gonorrhoeae* and rising gonorrhoea rates underscore the importance of optimising all tools that prevent and treat infection. The results here suggest reserve strategies are least optimal and favour combination therapy, although it will be important to revisit these conclusions with updated estimates of the rates of emergence of resistance and fitness cost of resistance, as well as with the effect of additional interventions as they become available.

## Supplementary Material

Supplementary Material

## Figures and Tables

**Table 1:** Model parameters

	Value	Domain	Source(s)
**Model calibration target**			
Gonorrhoea prevalence at start (calibration target mean)	0·03 (ie, 3·0%)	(0,1)	..
**Model population, sexual behaviour parameters**			
N, population size	10^6^	(0,¥)	Assumption
n, relative size of sexual activity groups	..	..	Tuite et al, 2017^17^
Low	n_1_=0·3	(0,1)	..
Intermediate	n_2_=0·6	(0,1)	..
High	n_3_=0·1	(0,1)	..
θ, rate of partner change per sexual activity group (per year)	..	..	Model fitting; Tuite et al, 2017^17^
Low (θ_1_)	1*1·22	(0,¥)	..
Intermediate (θ_2_)	5*1·22	(0,¥)	..
High (θ_3_)	20*1·22	(0,¥)	..
ε, mixing parameter	0·24	(0,1)	Model fitting; Tuite et al, 2017^17^
Proportion of cases drug A (ceftriaxone-like) resistant at start	0·0001	(0,1)	CDC GISP 2020;^12^ Tuite et al, 2017^17^
Proportion of cases drug B (new drug) resistant at start	0	(0,1)	Assumption
ρ, model entry or exit rate (per year)	1/20	(0,¥)	Tuite et al, 2017^17^
**Gonorrhoea natural history parameters**			
σ, proportion of incident infections that are symptomatic	0·60	(0,1)	Model fitting; Tuite et al, 2017^17^
b, transmission probability per partnership	0·46	(0,1)	Model fitting; Fingerhuth et al, 2016;^15^ Tuite et al, 2017;^17^ Tuite et al, 2018^18^
δ, natural recovery rate from infection (per year)	1/0·462	(0,¥)	Model fitting; Tuite et al,2017;^17^ Vegvari et al, 2020^19^
**Treatment parameters**			
T_s_, treatment rate if initial treatment success, symptomatic infection (per year)	1/0·031	(0,¥)	Model fitting; Tuite et al, 2017;^17^ Tuite et al, 2018^18^
T_sr_, treatment rate if initial treatment failure (requiring retreatment), symptomatic infection (per year)	T_s_/3	(0,¥)	Model fitting; Tuite et al, 2017^17^
T_m_, screening rate, asymptomatic infection (per year)	0·40	(0,¥)	Model fitting; Tuite et al, 2017;^17^ Tuite et al 2018;^18^ Hui et al, 2013^20^
ξ, probability of receiving drug upon initial treatment			
Assumption			
Drug A (ξ_A_)	Strategy dependent	(0,1)	..
Drug B (ξ_B_)	Strategy dependent	(0,1)	..
ω, probability of emergence of resistance upon treatment			
Drug A (ω_A_)	10^−8^	(0,1)	Tuite et al, 2017;^17^ Vegvari et al, 2020^19^
Drug B (ω_B_)	10^−4^ (10^−10^–10^−2^)	(0,1)	Assumption (range)
Drugs A and B (ω_AB_)	ω_A_*ω_B_	(0,1)	Assumption
f, relative fitness of resistant bacteria, compared to susceptible			
A resistant (f_A_)	0·98	(0,1)	Tuite et al, 2017^17^
B resistant (f_B_)	0·95 (0·80–1)	(0,1)	Assumption (range)
Dual A and B resistance (f_AB_)	f_A_*f_B_	(0,1)	Assumption
k_s_, proportion receiving retreatment if initial treatment failure, symptomatic infection	0·90	(0,1)	Tuite et al, 2017^17^

Parameters determined through model fitting might also cite previous literature sources, which were used to inform starting values for the maximum likelihood estimation procedure. CDC GISP=US Centers for Disease Control and Prevention Gonococcal Isolate Surveillance Project. ¥=infinity.

**Table 2:** Time to predefined 1% and 5% resistance thresholds by antibiotic introduction strategy under baseline model conditions

	Time to 1% resistance threshold (years)			Time to 5% resistance threshold (years)			End of drugs’ lifespan (T_L_)	Prevalence of gonorrhoea at T_L_	Mean number of incident infections per year, t <T_L_ (range)
	Drug A	Drug B	Drugs A and B	Drug A	Drug B	Drugs A and B			
Random 50–50 allocation	15·0	16·3	17·2	18·7	19·2	19·5	19·2	3·16%	179 023 (178 005–182 998)
Combination therapy	17·7	17·7	17·7	19·9	19·9	19·9	19·9	3·14%	178 641 (177 998–181 731)
Reserve strategy	4·7	11·8	14·6	6·5	13·9	17·9	13·9	3·13%	180 084 (178 011–184 405)
Gradual switch to 50–50	7·9	15·6	15·8	13·6	18·2	18·2	18·2	3·36%	180 983 (178 011–191 703)
Monotherapy, drug A	4·7	NA	NA	6·5	NA	NA	6·5	3·15%	179 220 (178 011–182 179)

T_L_=time in years until both drugs A and B hit their 5% resistance thresholds, warranting new treatment recommendations. The range is bounded by the absolute minimum and maximum annual results. NA=not applicable.

## Data Availability

All code needed to simulate the data, run the model and analysis, and produce numeric output and figures are available at https://github.com/emreichert13/gc-antibioticintro.

## Research in context

### Evidence before this study

We searched Google Scholar for modelling studies published between database inception and December, 2022 that compared antibiotic introduction strategies with the purpose of mitigating antibiotic resistance, using one or a combination of key search terms (ie, “antibiotic introduction”, “treatment protocol”, “antibiotic/antimicrobial resistance”, “gonorrhea”) plus “modeling”, with no language restrictions. We found several mathematical modelling studies that compared allocation strategies for multiple antibiotics, most of which focused on hospital-acquired infections. However, few studies had explored this question for sexually transmitted infections, and none focused on the optimal introduction approach for a novel and first-in-class antibiotic.

### Added value of this study

Our modelling study focused specifically on comparing introduction strategies for a novel, first-in-class antibiotic for gonorrhoea treatment within the existing ceftriaxone monotherapy landscape. It provides evidence that is pertinent to treatment protocol development once an antibiotic candidate for gonorrhoea treatment gains regulatory approval.

### Implications of all the available evidence

Reserving or restricting a novel antibiotic until substantial resistance has arisen to the current first-line drug might not be the optimal strategy for slowing the emergence and spread of resistance. In our exploratory model, combination therapy was the optimal strategy for delaying antibiotic resistance in most scenarios.

## References

[1] US Centers for Disease Control and Prevention. Sexually transmitted infections treatment guidelines, 2021. https://www.cdc.gov/std/treatment-guidelines/gonorrhea-adults.htm (accessed Feb 14, 2023).

[2] Taylor SN, Marrazzo J, Batteiger BE, Single-dose zoliflodacin (ETX0914) for treatment of urogenital gonorrhea. N Engl J Med 2018; 379: 1835–45. 30403954 10.1056/NEJMoa1706988

[3] Taylor SN, Morris DH, Avery AK, Gepotidacin for the treatment of uncomplicated urogenital gonorrhea: a phase 2, randomized, dose-ranging, single-oral dose evaluation. Clin Infect Dis 2018; 67: 504–12. 29617982 10.1093/cid/ciy145 PMC6070052

[4] Bonhoeffer S, Lipsitch M, Levin BR. Evaluating treatment protocols to prevent antibiotic resistance. Proc Natl Acad Sci USA 1997; 94: 12106–11. 9342370 10.1073/pnas.94.22.12106 PMC23718

[5] Bergstrom CT, Lo M, Lipsitch M. Ecological theory suggests that antimicrobial cycling will not reduce antimicrobial resistance in hospitals. Proc Natl Acad Sci USA 2004; 101: 13285–90. 15308772 10.1073/pnas.0402298101 PMC516561

[6] Wang YC, Lipsitch M. Upgrading antibiotic use within a class: tradeoff between resistance and treatment success. Proc Natl Acad Sci USA 2006; 103: 9655–60. 16772381 10.1073/pnas.0600636103 PMC1480462

[7] Haber M, Levin BR, Kramarz P. Antibiotic control of antibiotic resistance in hospitals: a simulation study. BMC Infect Dis 2010; 10: 254. 20738872 10.1186/1471-2334-10-254 PMC2940903

[8] Joyner ML, Manning CC, Canter BN. Modeling the effects of introducing a new antibiotic in a hospital setting: a case study. Math Biosci Eng 2012; 9: 601–25. 22881028 10.3934/mbe.2012.9.601

[9] Obolski U, Stein GY, Hadany L. Antibiotic restriction might facilitate the emergence of multi-drug resistance. PLoS Comput Biol 2015; 11: e1004340. 26110266 10.1371/journal.pcbi.1004340 PMC4481510

[10] Tepekule B, Uecker H, Derungs I, Frenoy A, Bonhoeffer S. Modeling antibiotic treatment in hospitals: a systematic approach shows benefits of combination therapy over cycling, mixing, and mono-drug therapies. PLoS Comput Biol 2017; 13: e1005745. 28915236 10.1371/journal.pcbi.1005745 PMC5600366

[11] Xiridou M, Soetens LC, Koedijk FDH, van der Sande MAB, Wallinga J. Public health measures to control the spread of antimicrobial resistance in *Neisseria gonorrhoeae* in men who have sex with men. Epidemiol Infect 2015; 143: 1575–84. 25275435 10.1017/S0950268814002519 PMC9507228

[12] US Centers for Disease Control and Prevention. Sexually transmitted disease surveillance 2020: Gonococcal Isolate Surveillance Project (GISP) profiles. https://www.cdc.gov/std/statistics/gisp-profiles/default.htm (accessed Feb 14, 2023).

[13] US Centers for Disease Control and Prevention. Sexually transmitted disease surveillance 2020. https://www.cdc.gov/std/statistics/2020/overview.htm (accessed Feb 14, 2023).

[14] US Centers for Disease Control and Prevention. Increases in fluoroquinolone-resistant *Neisseria gonorrhoeae* among men who have sex with men—United States, 2003, and revised recommendations for gonorrhea treatment, 2004. MMWR Morb Mortal Wkly Rep 2004; 53: 335–38. 15123985

[15] Fingerhuth SM, Bonhoeffer S, Low N, Althaus CL. Antibiotic-resistant *Neisseria gonorrhoeae* spread faster with more treatment, not more sexual partners. PLoS Pathog 2016; 12: e1005611. 27196299 10.1371/journal.ppat.1005611 PMC4872991

[16] Fingerhuth SM, Low N, Bonhoeffer S, Althaus CL. Detection of antibiotic resistance is essential for gonorrhoea point-of-care testing: a mathematical modelling study. BMC Med 2017; 15: 142. 28747205 10.1186/s12916-017-0881-x PMC5530576

[17] Tuite AR, Gift TL, Chesson HW, Hsu K, Salomon JA, Grad YH. Impact of rapid susceptibility testing and antibiotic selection strategy on the emergence and spread of antibiotic resistance in gonorrhea. J Infect Dis 2017; 216: 1141–49. 28968710 10.1093/infdis/jix450 PMC5853443

[18] Tuite AR, Rönn MM, Wolf EE, Estimated impact of screening on gonorrhea epidemiology in the United States: insights from a mathematical model. Sex Transm Dis 2018; 45: 713–22. 29894368 10.1097/OLQ.0000000000000876 PMC6813831

[19] Vegvari C, Grad YH, White PJ, Using rapid point-of-care tests to inform antibiotic choice to mitigate drug resistance in gonorrhoea. Eurosurveillance 2020; 25: 1900210. 33124551 10.2807/1560-7917.ES.2020.25.43.1900210 PMC7596916

[20] Hui BB, Wilson DP, Ward JS, The potential impact of new generation molecular point-of-care tests on gonorrhoea and chlamydia in a setting of high endemic prevalence. Sex Health 2013; 10: 348–56. 23806149 10.1071/SH13026

[21] Johnson Jones ML, Chapin-Bardales J, Bizune D, Extragenital chlamydia and gonorrhea among community venue-attending men who have sex with men—five cities, United States, 2017. MMWR Morb Mortal Wkly Rep 2019; 68: 321–25. 30973847 10.15585/mmwr.mm6814a1 PMC6459584

[22] Grov C, Cain D, Rendina HJ, Ventuneac A, Parsons JT. Characteristics associated with urethral and rectal gonorrhea and chlamydia diagnoses in a US national sample of gay and bisexual men: results from the one thousand strong panel. Sex Transm Dis 2016; 43: 165–71. 26859803 10.1097/OLQ.0000000000000410 PMC4748382

[23] Bolker B, R Development Core Team. bbmle: tools for general maximum likelihood estimation. R package version 1.0.25. https://CRAN.R-project.org/package=bbmle (accessed June 1, 2022).

[24] Jacobsson S, Golparian D, Oxelbark J, Pharmacodynamic evaluation of zoliflodacin treatment of *Neisseria gonorrhoeae* strains with amino acid substitutions in the zoliflodacin target GyrB using a dynamic hollow fiber infection model. Front Pharmacol 2022; 13: 874176. 35496288 10.3389/fphar.2022.874176 PMC9046595

[25] Tapsall J, Anti-Infective Drug Resistance Surveillance and Containment Team. Antimicrobial resistance in *Neisseria gonorrhoeae*. World Health Organization; 2001, report no: WHO/CDS/CSR/DRS/2001.3. https://apps.who.int/iris/handle/10665/66963 (accessed Feb 14, 2023).

[26] Soetaert K, Petzoldt T, Setzer R. Solving differential equations in R: Package deSolve. J Stat Softw 2010; 33: 1–25.20808728

[27] Olesen SW, Grad YH. Deciphering the impact of bystander selection for antibiotic resistance in *Neisseria gonorrhoeae*. J Infect Dis 2020; 221: 1033–35. 30957162 10.1093/infdis/jiz156 PMC7360351

[28] Adamson PC, Lin EY, Ha SM, Klausner JD. Using a public database of *Neisseria gonorrhoeae* genomes to detect mutations associated with zoliflodacin resistance. J Antimicrob Chemother 2021; 76: 2847–49. 34324655 10.1093/jac/dkab262 PMC8521401

[29] Scangarella-Oman NE, Hossain M, Dixon PB, Microbiological analysis from a phase 2 randomized study in adults evaluating single oral doses of gepotidacin in the treatment of uncomplicated urogenital gonorrhea caused by *Neisseria gonorrhoeae*. Antimicrob Agents Chemother 2018; 62: e01221–18. 30249694 10.1128/AAC.01221-18 PMC6256812

