# Supplementary material for: Resistance-minimising strategies for introducing a novel antibiotic for gonorrhoea treatment: a mathematical modelling study
Source: Lancet Microbe. Author manuscript; Available in PMC 2024 Feb 14. (PMC10865326; doi:10.1016/S2666-5247(23)00145-3)
Supplement: Supplementary Material [file NIHMS1935464-supplement-Supplementary_Material.pdf]

THE LANCET
Microbe

Supplementary appendix

This appendix formed part of the original submission and has been peer reviewed. We post it as supplied by the authors.

Supplement to: Reichert E, Yaesoubi R, Rönn MM, Gift TL, Salomon JA, Grad YH. Resistance-minimising strategies for introducing a novel antibiotic for gonorrhoea treatment: a mathematical modelling study. *Lancet Microbe* 2023; published online Aug 21. https://doi.org/10.1016/S2666-5247(23)00145-3.

Supplementary Material

Supplementary Figure 1. Sigmoid functions that define probability of treatment with drugs A and B (ξ_A, ξ_B) under the A) reserve and B) gradual switch strategies. 2

Supplementary Figure 2. Percentage of gonococcal infections with each resistance profile over time, by strategy. 3

Supplementary Figure 3. Dynamics of A) incidence rate ratios and B) cumulative number of incident infections averted over time, relative to the reserve strategy. 4

Supplementary Figure 4. Additional time (in years) to loss of each of antibiotics A and B by strategy relative to that of the reserve strategy (T_L|strategy - T_L|reserve), based on properties of a new antibiotic B. 5

Supplementary Table 1. Time to predefined 5% resistance threshold by antibiotic introduction strategy under baseline model conditions for two alternative mean calibration targets (1.5% and 6.0% gonorrhea prevalence). 6

Technical Supplement 7

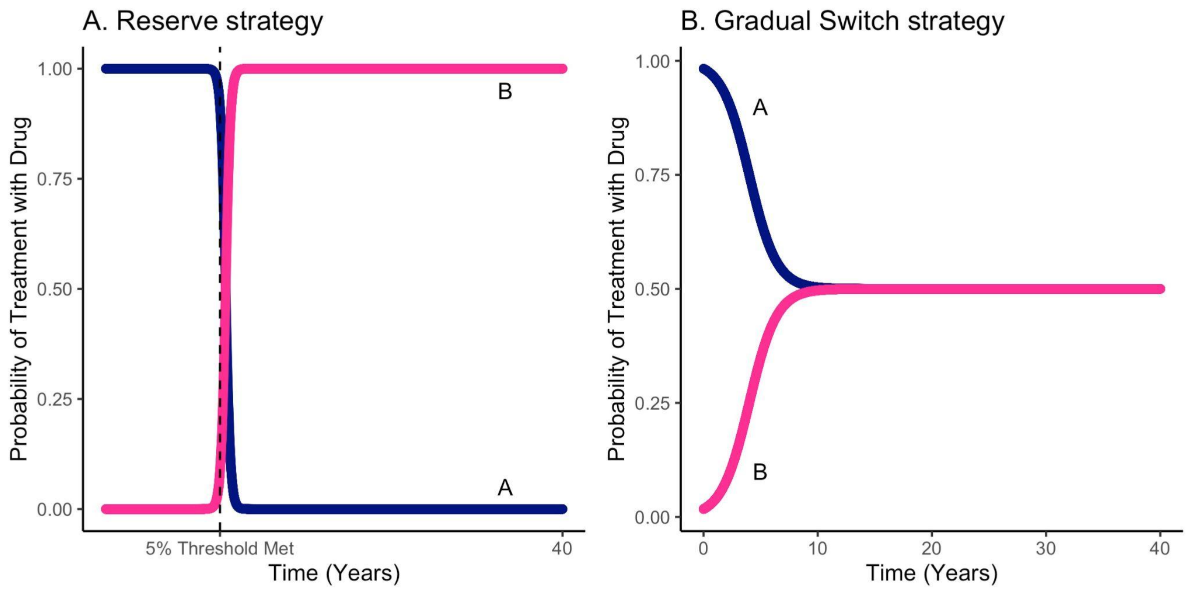

Supplementary Figure 1. Sigmoid functions that define probability of treatment with drugs A and B (ξ_A, ξ_B) under the A) reserve and B) gradual switch strategies. A) The probability of receiving each drug is updated over time; a change is triggered at the time drug A reaches the 5% prevalence of resistance threshold. As drug A resistance hits this threshold, drug B is quickly phased in from 0% use to 100% use with a midpoint 0.5 years beyond the point at which drug A is lost. B) The probability of receiving each drug is updated over time. Drug B is phased in from a small initial uptake at time 0 (1.7%) to 50% use with a midpoint of 4 years. Complete random 50-50 allocation is reached within 9.5 years.

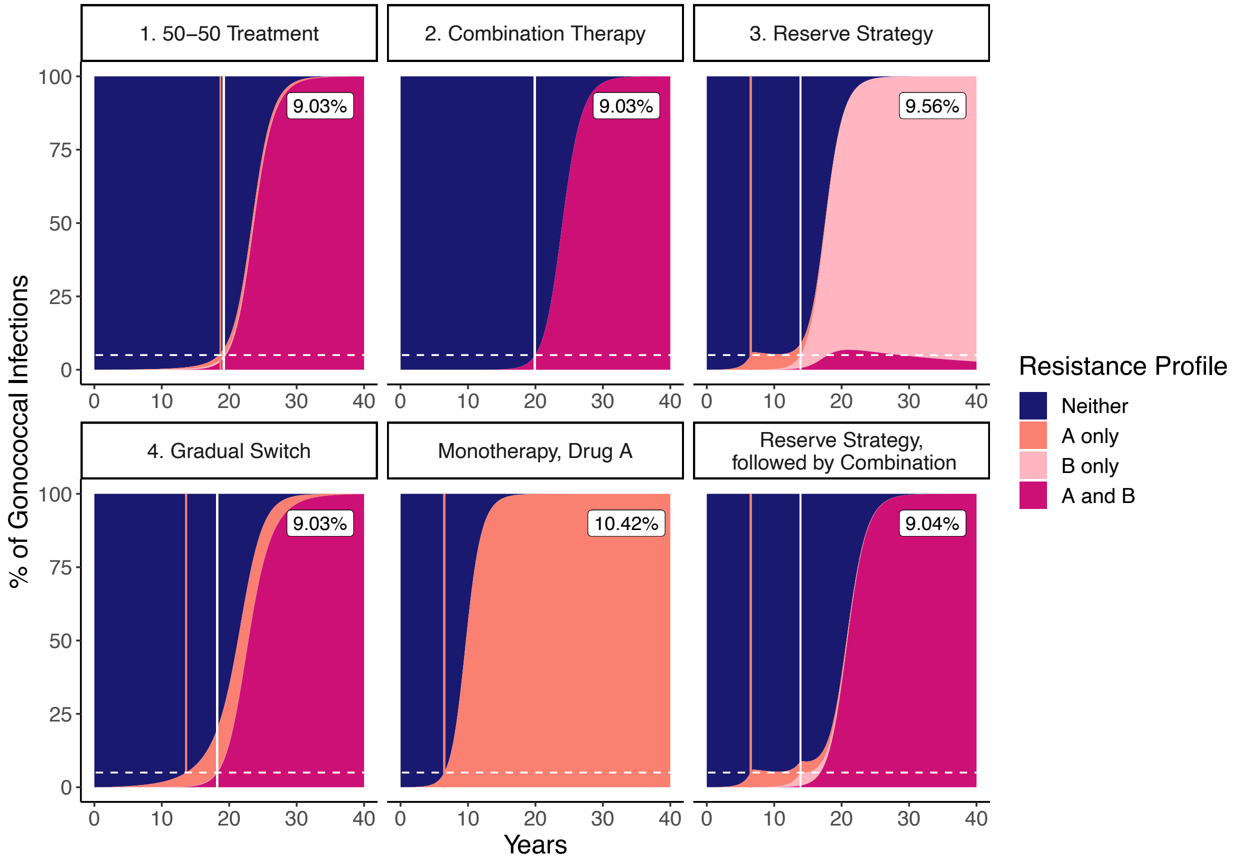

Supplementary Figure 2. Percentage of gonococcal infections with each resistance profile over time, by strategy. The four antibiotic allocation strategies of interest are visualized along with i) continuing monotherapy using only drug A and ii) deploying combination therapy after each of drugs A and B are lost under the reserve strategy, for comparison. Dotted horizontal white lines indicate the 5% prevalence of resistance threshold, which warrants changes in treatment protocols per WHO guidelines. Solid vertical lines delineate the time at which only drug A is lost (salmon) and both drugs are lost (T_L; white) by strategy. The population-level prevalence of gonococcal infection that the model re-equilibrates to is labeled in the upper right corner for each strategy.

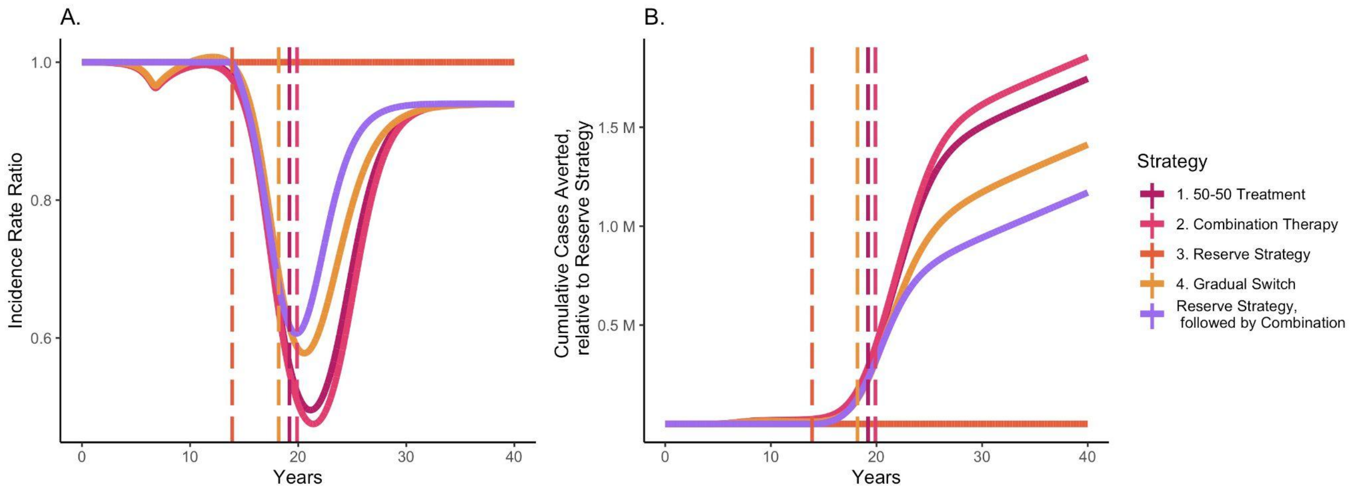

Supplementary Figure 3. Dynamics of A) incidence rate ratios and B) cumulative number of incident infections averted over time, relative to the reserve strategy. Dashed vertical lines represent the end of the drugs' lifespan (T_L) for each introduction strategy under baseline model assumptions.

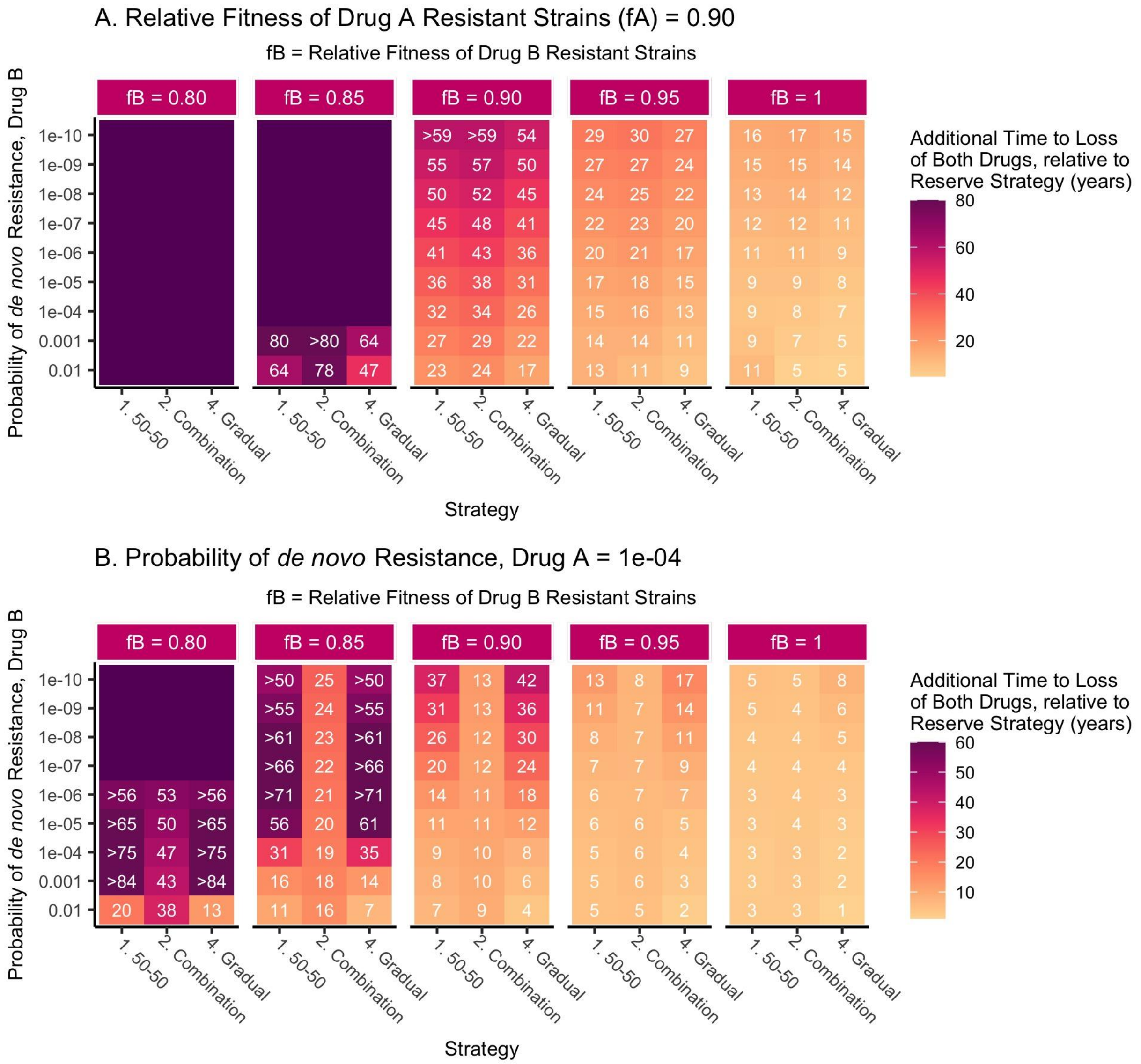

Supplementary Figure 4. Additional time (in years) to loss of each of antibiotics A and B by strategy relative to that of the reserve strategy (T_L|strategy - T_L|reserve), based on properties of a new antibiotic B. Antibiotic A = ceftriaxone-like, Antibiotic B = new antibiotic. Strategies (x-axis) are compared over a range of plausible values for the probability of *de novo* resistance (or emergence of resistance upon treatment) with drug B (ω_B; y-axis) and the relative fitness of drug B resistant strains (f_B; vertical facets). **A)** The fitness cost associated with resistance to drug A is increased to 0.10. **B)** The probability of resistance upon treatment with drug A is increased to 10^{-4}. The model run time was extended to 100 years because some parameter sets increased the lifespan of available antibiotics >40 years for all strategies. If the lifespan of the drugs extended beyond 100 years, that strategy's results are shown either in relative terms for comparison or with an unlabeled dark purple tile, if no strategies on the x-axis had a defined T_L under that parameter set for drug B. Abbreviations: fB = f_B = fitness of drug B resistant strains relative to susceptible bacteria, fA = f_A = fitness of drug A resistant strains relative to susceptible bacteria, T_L = time in years until both drugs A and B have hit their 5% resistance thresholds, warranting new treatment recommendations.

Supplementary Table 1. Time to predefined 5% resistance threshold by antibiotic introduction strategy under baseline model conditions for two alternative mean calibration targets (1.5% and 6.0% gonorrhea prevalence). Baseline parameters for drugs A and B are assumed. Model recalibration alters parameter values for each of the eight parameters determined through model fitting as identified in Table 1. T_L = time in years until both drugs A and B hit their 5% resistance thresholds, warranting new treatment recommendations.

	**1.5% prevalence calibration target**					**6.0% prevalence calibration target**				
	Time to 5% Resistance Threshold (years)			End of Drugs' Lifespan (T_L)	Prevalence of Gonorrhea at T_L	Time to 5% Resistance Threshold (years)			End of Drugs' Lifespan (T_L)	Prevalence of Gonorrhea at T_L
	Drug A	*Drug B*	*Dual*			*Drug A*	*Drug B*	*Dual*		
1. Random 50-50 Allocation	22.3	23.1	23.4	23.1	2.0%	15.2	15.4	15.5	15.4	6.1%
2. Combination therapy	24.0	24.0	24.0	24.0	2.0%	15.9	15.9	15.9	15.9	6.1%
3. Reserve strategy	7.7	16.5	22.0	16.5	2.0%	5.2	11.2	14.0	11.2	6.0%
4. Gradual Switch to 50-50	16.8	21.8	21.9	21.8	2.1%	10.2	14.2	14.2	14.2	6.3%
Monotherapy, Drug A	7.6	*NA*	*NA*	7.6	1.9%	5.2	*NA*	*NA*	5.2	6.1%

Technical Supplement

Model Structure

We use an adapted version of the single sex compartmental gonorrhea transmission model developed by Tuite et al.[1] to describe our two-drug system. A visual overview of the model is presented in the main text (**Figure 1**). As previously described[1], the model population is stratified into three sexual activity groups (k; low, intermediate, and high), characterized by annual rates of partner change. The model can be characterized as a susceptible-infectious-susceptible (SIS) model, where susceptible individuals (S) become infected and can then recover spontaneously or through antibiotic treatment. For those that seek treatment, one can receive drug A (ceftriaxone-like) and/or drug B (new antibiotic). Infections (I) are stratified by symptomatic (Y) versus asymptomatic (Z) infection, as well as by resistance profile, where each infection can be caused by bacteria resistant to the ceftriaxone-like drug A (Y_{Ak}, Z_{Ak}), the novel drug B (Y_{Bk}, Z_{Bk}), neither (Y_{0k}, Z_{0k}), or both (Y_{ABk}, Z_{ABk}). The total size of the population (N) is set at 10^6 and the absolute size of each sexual activity group at N_k, with the relative size of each group fixed at n_k. Therefore,

$$N_k = S_k + I_k, \text{ where } I_k = Y_{0k} + Y_{Ak} + Y_{Bk} + Y_{ABk} + Z_{0k} + Z_{Ak} + Z_{Bk} + Z_{ABk}$$

The relative rate of partner change (r_k) for each sexual activity group is drawn from previous estimates by Tuite et al.[1] determined by data from the National HIV Behavioral Surveillance System[2]. The rate of partner change (c_{min}) in the low activity group is estimated through the maximum likelihood estimation model fitting procedure. The annual rate of partner change for each activity group (θ_k) is therefore described by the equation:

$$\theta_k = r_k * c_{min}$$

Assortativity between sexual activity groups is characterized by mixing parameter ε [which can range from 0 (random mixing) and 1 (fully assortative mixing) and is determined through model fitting]. This leads the probability of an individual from sexual activity group i coming into sexual contact with an individual from group j to be:

$$p_{ij} = \varepsilon x_{ij}\frac{\theta_i}{N_i} + (1-\varepsilon)\frac{\theta_i \theta_j}{\sum_{k=1}^{3} \theta_k N_k}$$

where x_{ij} is equal to 1 if $i = j$ and equal to 0 if $i \neq j$. The rate of infection for susceptible individuals in sexual activity group i from infected partners of group j ($\beta_{i \leftarrow j}$) is proportional to this per capita probability of sexual contact between groups i and j (p_{ij}), as well as the transmission probability per partnership (b). This relationship is defined by:

$$\beta_{i \leftarrow j} = b p_{ij}$$

Therefore, the per capita transmission matrix $\boldsymbol{\beta}$ is a $k \times k$ matrix with elements $\beta_{i \leftarrow j}$ at row i and column j, defined as the per capita rate of gonorrhea transmission to group i from group j. Note that the transmission matrix at element $\beta_{i \leftarrow j}$ is divided through by the size of group j's population, which stays constant over time. As area is also held constant, and deemed not influential in per capita transmission rates, the model assumes constant density and yields results identical to frequency-dependent transmission.

Model Recovery from Gonococcal Infection

Parameters describing treatment and retreatment rates by infection type (symptomatic vs. asymptomatic), as well as the rate of natural clearance of infection, are presented in the main text (Table 1). These parameters can be used to calculate the average duration of an infection (years), which is ($1/T_s$) for treated symptomatic infections, ($1/T_m$) for treated asymptomatic infections detected via screening, and ($1/\delta$) for infections that clear naturally. The treatment rate T_{sr} for those with an initial treatment failure assumes an average duration of infection three times that of those with initial treatment success; $1/T_{sr}$ represents the average time in years until successful retreatment, including the time it takes for: 1) the individual to receive the initial failed treatment, 2) the individual to re-seek care, and 3) the provider to identify and prescribe the correct antibiotic for retreatment. It is possible for resistance acquired upon initial treatment of a susceptible infection to then be retreated insufficiently with the same antibiotic before receiving a successful retreatment; in these instances, the average duration of infection is ($1/T_s + 1/T_{sr}$), in years.

Model Equations

In matrix form, the model is described by the following system of differential equations, where $\circ$ denotes element-wise multiplication. All parameters are defined in the main text (**Table 1**). Bolded letters represent $k \times 1$ column vectors with compartmental variables for each sexual activity group (e.g., $\mathbf{S} = [S_1,...S_k]^T$) for k groups), with the exception of the $k \times k$ matrix $\boldsymbol{\beta}$.

Strategies 1,3,4: Random (50-50) allocation, reserve strategy, and gradual introduction:

$$
\begin{aligned}
d\mathbf{S}/dt = & -\boldsymbol{\beta}((\mathbf{Y}_0 + \mathbf{Z}_0) + f_A(\mathbf{Y}_A + \mathbf{Z}_A) + f_B(\mathbf{Y}_B + \mathbf{Z}_B) + f_{AB}(\mathbf{Y}_{AB} + \mathbf{Z}_{AB}))\circ\mathbf{S} + \\
& (1- \xi_A\omega_A - \xi_B\omega_B)(T_s\mathbf{Y}_0 + T_m\mathbf{Z}_0) + \\
& \xi_B(1-\omega_B)(T_s\mathbf{Y}_A + T_m\mathbf{Z}_A) + \\
& \xi_A(1-\omega_A)(T_s\mathbf{Y}_B + T_m\mathbf{Z}_B) + \\
& (1-\xi_B)(1-\omega_B)\kappa_s T_{sr}\mathbf{Y}_A + \\
& (1-\xi_A)(1-\omega_A)\kappa_s T_{sr}\mathbf{Y}_B + \\
& \kappa_s T_{sr}\mathbf{Y}_{AB} + \\
& \delta(\mathbf{Y}_0 + \mathbf{Y}_A + \mathbf{Y}_B + \mathbf{Y}_{AB} + \mathbf{Z}_0 + \mathbf{Z}_A + \mathbf{Z}_B + \mathbf{Z}_{AB}) + \\
& \rho\mathbf{N} - \rho\mathbf{S}
\end{aligned}
$$

$d\mathbf{Y}_0/dt = \sigma\boldsymbol{\beta}(\mathbf{Y}_0 + \mathbf{Z}_0)\circ\mathbf{S} - T_s\mathbf{Y}_0 - \delta\mathbf{Y}_0 - \rho\mathbf{Y}_0$
$d\mathbf{Z}_0/dt = (1\text{-}\sigma)\boldsymbol{\beta}(\mathbf{Y}_0 + \mathbf{Z}_0)\circ\mathbf{S} - T_m\mathbf{Z}_0 - \delta\mathbf{Z}_0 - \rho\mathbf{Z}_0$

$d\mathbf{Y}_A/dt = \sigma f_A\boldsymbol{\beta}(\mathbf{Y}_A + \mathbf{Z}_A)\circ\mathbf{S} + \xi_A\omega_A T_s\mathbf{Y}_0 - \xi_B T_s\mathbf{Y}_A - (1\text{-}\xi_B)\kappa_s T_{sr}\mathbf{Y}_A - \delta\mathbf{Y}_A - \rho\mathbf{Y}_A$
$d\mathbf{Z}_A/dt = (1\text{-}\sigma)f_A\boldsymbol{\beta}(\mathbf{Y}_A + \mathbf{Z}_A)\circ\mathbf{S} + \xi_A\omega_A T_m\mathbf{Z}_0 - \xi_B T_m\mathbf{Z}_A - \delta\mathbf{Z}_A - \rho\mathbf{Z}_A$

$d\mathbf{Y}_B/dt = \sigma f_B\boldsymbol{\beta}(\mathbf{Y}_B + \mathbf{Z}_B)\circ\mathbf{S} + \xi_B\omega_B T_s\mathbf{Y}_0 - \xi_A T_s\mathbf{Y}_B - (1\text{-}\xi_A)\kappa_s T_{sr}\mathbf{Y}_B - \delta\mathbf{Y}_B - \rho\mathbf{Y}_B$
$d\mathbf{Z}_B/dt = (1\text{-}\sigma)f_B\boldsymbol{\beta}(\mathbf{Y}_B + \mathbf{Z}_B)\circ\mathbf{S} + \xi_B\omega_B T_m\mathbf{Z}_0 - \xi_A T_m\mathbf{Z}_B - \delta\mathbf{Z}_B - \rho\mathbf{Z}_B$

$d\mathbf{Y}_{AB}/dt = \sigma f_{AB}\boldsymbol{\beta}(\mathbf{Y}_{AB} + \mathbf{Z}_{AB})\circ\mathbf{S} + \xi_A\omega_A T_s\mathbf{Y}_B + \xi_B\omega_B T_s\mathbf{Y}_A + (1\text{-}\xi_B)\omega_B\kappa_s T_{sr}\mathbf{Y}_A + (1\text{-}\xi_A)\omega_A\kappa_s T_{sr}\mathbf{Y}_B -$
$\kappa_s T_{sr}\mathbf{Y}_{AB} - \delta\mathbf{Y}_{AB} - \rho\mathbf{Y}_{AB}$
$d\mathbf{Z}_{AB}/dt = (1\text{-}\sigma)f_{AB}\boldsymbol{\beta}(\mathbf{Y}_{AB} + \mathbf{Z}_{AB})\circ\mathbf{S} + \xi_A\omega_A T_m\mathbf{Z}_B + \xi_B\omega_B T_m\mathbf{Z}_A - \delta\mathbf{Z}_{AB} - \rho\mathbf{Z}_{AB}$

Strategy 2: Combination treatment:
Note: ξ_A and ξ_B are omitted from the following equations since both = 1.

$d\mathbf{S}/dt = -\boldsymbol{\beta}((\mathbf{Y}_0 + \mathbf{Z}_0) + f_A(\mathbf{Y}_A + \mathbf{Z}_A) + f_B(\mathbf{Y}_B + \mathbf{Z}_B) + f_{AB}(\mathbf{Y}_{AB} + \mathbf{Z}_{AB}))\circ\mathbf{S} +$
$(1\text{-} \omega_A\omega_B)(T_S\mathbf{Y}_0 + T_m\mathbf{Z}_0) +$
$(1\text{-}\omega_B)(T_S\mathbf{Y}_A + T_m\mathbf{Z}_A) +$
$(1\text{-}\omega_A)(T_S\mathbf{Y}_B + T_m\mathbf{Z}_B) +$
$\kappa_s T_{sr}\mathbf{Y}_{AB} +$
$\delta(\mathbf{Y}_0 + \mathbf{Y}_A + \mathbf{Y}_B + \mathbf{Y}_{AB} + \mathbf{Z}_0 + \mathbf{Z}_A + \mathbf{Z}_B + \mathbf{Z}_{AB}) +$
$\rho\mathbf{N} - \rho\mathbf{S}$

$d\mathbf{Y}_0/dt = \sigma\boldsymbol{\beta}(\mathbf{Y}_0 + \mathbf{Z}_0)\circ\mathbf{S} - T_s\mathbf{Y}_0 - \delta\mathbf{Y}_0 - \rho\mathbf{Y}_0$
$d\mathbf{Z}_0/dt = (1\text{-}\sigma)\boldsymbol{\beta}(\mathbf{Y}_0 + \mathbf{Z}_0)\circ\mathbf{S} - T_m\mathbf{Z}_0 - \delta\mathbf{Z}_0 - \rho\mathbf{Z}_0$

$d\mathbf{Y}_A/dt = \sigma f_A\boldsymbol{\beta}(\mathbf{Y}_A + \mathbf{Z}_A)\circ\mathbf{S} - T_s\mathbf{Y}_A - \delta\mathbf{Y}_A - \rho\mathbf{Y}_A$
$d\mathbf{Z}_A/dt = (1\text{-}\sigma)f_A\boldsymbol{\beta}(\mathbf{Y}_A + \mathbf{Z}_A)\circ\mathbf{S} - T_m\mathbf{Z}_A - \delta\mathbf{Z}_A - \rho\mathbf{Z}_A$

$d\mathbf{Y}_B/dt = \sigma f_B\boldsymbol{\beta}(\mathbf{Y}_B + \mathbf{Z}_B)\circ\mathbf{S} - T_s\mathbf{Y}_B - \delta\mathbf{Y}_B - \rho\mathbf{Y}_B$
$d\mathbf{Z}_B/dt = (1\text{-}\sigma)f_B\boldsymbol{\beta}(\mathbf{Y}_B + \mathbf{Z}_B)\circ\mathbf{S} - T_m\mathbf{Z}_B - \delta\mathbf{Z}_B - \rho\mathbf{Z}_B$

$d\mathbf{Y}_{AB}/dt = \sigma f_{AB}\boldsymbol{\beta}(\mathbf{Y}_{AB} + \mathbf{Z}_{AB})\circ\mathbf{S} + \omega_A T_s\mathbf{Y}_B + \omega_B T_s\mathbf{Y}_A + \omega_A\omega_B T_s\mathbf{Y}_0 - \kappa_s T_{sr}\mathbf{Y}_{AB} - \delta\mathbf{Y}_{AB} - \rho\mathbf{Y}_{AB}$
$d\mathbf{Z}_{AB}/dt = (1\text{-}\sigma)f_{AB}\boldsymbol{\beta}(\mathbf{Y}_{AB} + \mathbf{Z}_{AB})\circ\mathbf{S} + \omega_A T_m\mathbf{Z}_B + \omega_B T_m\mathbf{Z}_A + \omega_A\omega_B T_m\mathbf{Z}_0 - \delta\mathbf{Z}_{AB} - \rho\mathbf{Z}_{AB}$

We calculate the number of incident infections at time *t*, or the overall force of infection across sexual activity groups, as $\lambda_t = \boldsymbol{\beta}((\mathbf{Y}_{0t} + \mathbf{Z}_{0t}) + f_A(\mathbf{Y}_{At} + \mathbf{Z}_{At}) + f_B(\mathbf{Y}_{Bt} + \mathbf{Z}_{Bt}) + f_{AB}(\mathbf{Y}_{ABt} + \mathbf{Z}_{ABt}))\circ\mathbf{S}_t$.

The overall prevalence of infection at time t can be calculated as $\text{Prev}_t = (\mathbf{Y}_{0t} + \mathbf{Z}_{0t} + \mathbf{Y}_{At} + \mathbf{Z}_{At} + \mathbf{Y}_{Bt} + \mathbf{Z}_{Bt} + \mathbf{Y}_{ABt} + \mathbf{Z}_{ABt})/N$.

References

1. Tuite AR, Gift TL, Chesson HW, Hsu K, Salomon JA, Grad YH. Impact of Rapid Susceptibility Testing and Antibiotic Selection Strategy on the Emergence and Spread of Antibiotic Resistance in Gonorrhea. *J Infect Dis*. 2017;216(9):1141-1149. doi:10.1093/infdis/jix450

2. Centers for Disease Control and Prevention. HIV Infection Risk, Prevention, and Testing Behaviors among Men Who Have Sex With Men -- National HIV Behavioral Surveillance, 20 U.S. Cities, 2014. HIV Surveillance Special Report 15. Published January 2016. Accessed September 8, 2022. http://www.cdc.gov/hiv/library/reports/surveillance/#panel2
